# Benthic microbial communities of coastal terrestrial and ice shelf Antarctic meltwater ponds

**DOI:** 10.3389/fmicb.2015.00485

**Published:** 2015-05-27

**Authors:** Stephen D. J. Archer, Ian R. McDonald, Craig W. Herbold, Charles K. Lee, Craig S. Cary

**Affiliations:** International Centre for Terrestrial Antarctic Research, School of Science, University of WaikatoHamilton, New Zealand

**Keywords:** biogeography, Antarctic, benthic, microbial, pond

## Abstract

The numerous perennial meltwater ponds distributed throughout Antarctica represent diverse and productive ecosystems central to the ecological functioning of the surrounding ultra oligotrophic environment. The dominant taxa in the pond benthic communities have been well described however, little is known regarding their regional dispersal and local drivers to community structure. The benthic microbial communities of 12 meltwater ponds in the McMurdo Sound of Antarctica were investigated to examine variation between pond microbial communities and their biogeography. Geochemically comparable but geomorphologically distinct ponds were selected from Bratina Island (ice shelf) and Miers Valley (terrestrial) (<40 km between study sites), and community structure within ponds was compared using DNA fingerprinting and pyrosequencing of 16S rRNA gene amplicons. More than 85% of total sequence reads were shared between pooled benthic communities at different locations (OTU_0.05_), which in combination with favorable prevailing winds suggests aeolian regional distribution. Consistent with previous findings *Proteobacteria* and *Bacteroidetes* were the dominant phyla representing over 50% of total sequences; however, a large number of other phyla (21) were also detected in this ecosystem. Although dominant Bacteria were ubiquitous between ponds, site and local selection resulted in heterogeneous community structures and with more than 45% of diversity being pond specific. Potassium was identified as the most significant contributing factor to the cosmopolitan community structure and aluminum to the location unique community based on a BEST analysis (Spearman's correlation coefficient of 0.632 and 0.806, respectively). These results indicate that the microbial communities in meltwater ponds are easily dispersed regionally and that the local geochemical environment drives the ponds community structure.

## Introduction

Antarctic aquatic mats and associated sediments harbor diverse microbial communities crucial to nutrient cycling (Bowman et al., 2000) and are the presumed dominant source of terrestrial biomass outside of coastal areas (Moorhead et al., 2003; Wood et al., 2008). Although smaller than their well-studied lake counterparts, meltwater ponds derived from local ice and snow melt, are more abundant throughout the continent, providing individually distinct geochemical environments (Matsumoto et al., 1992; Doran et al., 1994; Vincent and James, 1996). Their significance to terrestrial Antarctic processes, coupled with a relatively high biodiversity, productivity and responsiveness to the geochemical environment (Sabbe et al., 2004; Jungblut et al., 2005; Sutherland, 2009; Safi et al., 2012), makes these ponds useful for monitoring future environmental changes in Antarctica. Polar pond mats and sediments have been investigated in the past (Suren, 1990; VanTrappen et al., 2002; de los Rios et al., 2004; Sabacka and Elster, 2006; Rojas et al., 2009; Sutherland, 2009), however few studies have utilized high-throughput sequencing to accurately describe and compare the bacterial community structure between ponds or locations.

**Figure 1:**
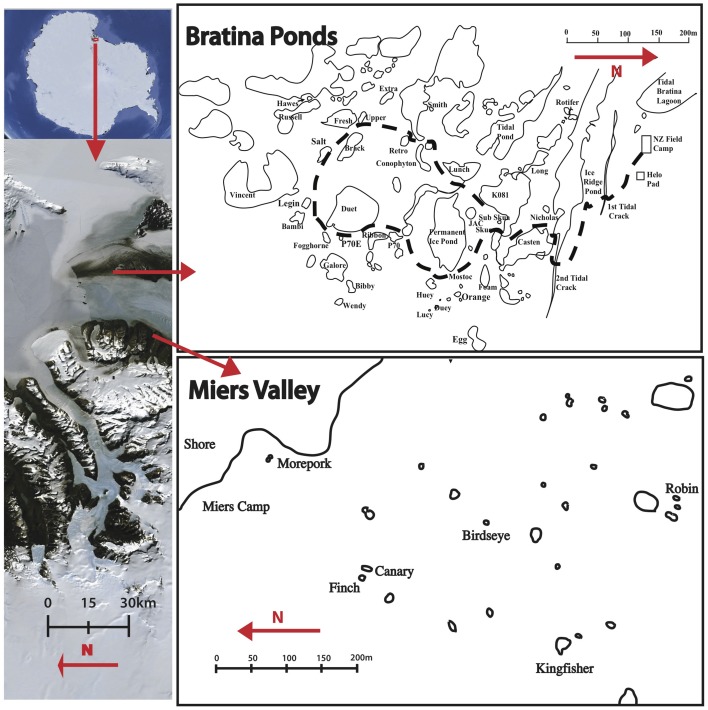
**Location of study sites in the Ross sea region of Antarctica (left), and the scale and proximity of ponds at Bratina Island (top right) and the Miers Valley (bottom right)**.

The meltwater ponds at Bratina Island, Victoria Land on the McMurdo Ice Shelf (MIS ponds) are probably the best characterized to date (Hawes et al., 2013). Bratina Island is located in the southwest corner of the Ross Sea, at the northern tip of Brown Peninsula with the coast of southern Victoria Land to the west (Figure 1). The undulating landscape, driven by compression of the ice shelf, provides an ideal terrain for the formation of thousands of meltwater ponds (Howard-Williams et al., 1990). A 10–30 cm layer of soil/sediment deposits covers this region, originating from basal freezing of marine sediments to the underside of the floating ice shelf and deposition by surface ablation (Kellogg and Kellogg, 1987, 1988). Within 1 km of an established field camp are a series of previously studied ponds of variable size, depth, age, and chemistry.

Although considered a polar desert, dozens of terrestrial meltwater ponds are distributed throughout the Dry Valleys of Antarctica (Vincent and James, 1996). The McMurdo Dry Valleys are the largest ice-free area of the Antarctic continent encompassing a series of valleys whose soil biological communities are influenced by local geology, chemistry and geographic factors (Cary et al., 2010). A number of poorly studied coastal terrestrial ponds (CT ponds) are located at the eastern most point (the “mouth”) of the Miers Valley. Although formed by meltwater accumulation in landscape depressions, as is seen at Bratina Island, the <30 cm soils in the Miers Valley cover continental permafrost and typically originate from bedrock and glacial till erosion (Healy et al., 2006; McLeod et al., 2009; Cary et al., 2010).

The cyanobacterial mats ubiquitous in Antarctic aquatic ecosystems are morphologically complex and variable, their structure dependent on local substrate composition (grain size, deposition rates etc.), the dominant *Cyanobacteria* present, climatological influences and pond geochemistry (de los Rios et al., 2004). As in temperate climates, the sediments underlying these pond mats (Mountfort et al., 2003) contain a diverse microbial community with vertical stratification defined by the immediate redox conditions (Ye et al., 2009; Shivaji et al., 2011). These sediments have built up over decades, even through thick ice cover, by repeated layering of aeolian dust from the surrounding environment and mat re-growth (Squyres et al., 1991). The visually dominant *Cyanobacteria* are well studied in Antarctic meltwater pond mats and sediments (Taton et al., 2003, 2006; Sabbe et al., 2004; Jungblut et al., 2005; Wood et al., 2008), however, *Proteobacteria* and *Bacteroidetes* are also consistently identified as significant components of the microbial community (Bowman et al., 2000; VanTrappen et al., 2002; Sjoling and Cowan, 2003; Tang et al., 2013) with other intermittently identified phyla such as *Firmicutes, Actinobacteria, Verrucomicrobia*, and *Acidobacteria* (Brambilla et al., 2001; Rojas et al., 2009; Peeters et al., 2012).

This study presents a comparative survey of the benthic communities from 12 meltwater ponds in the Ross Sea Region of Antarctica. By utilizing high-throughput sequencing coupled with biogeochemical data this represents the highest resolution comparison of these communities ever undertaken. As a significant legacy of research has been conducted on the cyanobacterial component of this community, primers were selected to preferentially target the non-cyanobacterial, bacterial component of the microbial community. The objectives of this study were to compare the bacterial benthic communities from MIS and CT ponds, to identify and describe the dominant phyla and OTUs across the ponds, and to investigate the geochemical drivers of community structure so that a greater understanding of these unique communities could be gained.

## Materials and methods

### Field sampling strategy

Sediment cores, four-centimeters in depth, were collected from around the edge of 12 fully thawed meltwater ponds during the summer season in January 2013 from Bratina Island (6 ponds) (78° 01′ S, 165° 32′ E) and the Miers Valley (6 ponds) (78° 07′ S, 164° 12′ E) (Table 1, Figure 1). Sites were selected to encompass a broad range of surface water geochemistry from ponds at each location. Cores were aseptically collected using a disposable push-corer developed from a 50 mL syringe (BD, Singapore). The corer (with the plunger removed) was inserted 4–6 cm into the sediment, the plunger reinserted and core removed carefully to retain the sediment structure. After excess sediment was removed, each core was sub-sectioned into four one-centimeter samples, placed in sterile 15 oz whirlpack (Nasco, WI, USA), then frozen for transportation to the laboratory.

**Table 1:** ***In situ* environmental data from overlying water column**.

**Pond**	**Location**	**GPS Coordinates**		**DO(ppm)**	**Conductivity(mS/cm)**	**pH**
**P70E**	Bratina	S 78.01580	E 165.55165	11.61	7.65	8.76
**Huey**	Bratina	S 78.01419	E 165.55576	13.08	4.35	9.48
**Legin**	Bratina	S 78.01624	E 165.54903	13.15	2.2	9.65
Salt	Bratina	S 78.01608	E 165.54510	14.81	40.8	9.58
Bambi	Bratina	S 78.01649	E 165.54936	14.79	3.31	10.55
Conophyton	Bratina	S 78.01431	E 165.54500	12.26	0.617	9.73
**Finch**	Miers	S 78 07.748	E 164 11.714	13.35	1.742	9.76
Birdseye	Miers			20.18	14.38	9.84
**Canary**	Miers	S 78 07.433	E 164 11.453	14.07	4.18	9.58
Robin	Miers	S 78 07.752	E 164 11.762	13.94	0.634	10.06
Kingfisher	Miers			14.02	0.987	9.39
**Morepork**	Miers	S 78 07.343	E 164 12.061	12.26	4.06	9.13

*Pond names in bold represent those selected for high-throughput sequencing*.

### Geochemical data

*In situ* dissolved oxygen, pH, temperature and conductivity of the overlying pond water were measured using an HQ40d portable multi-parameter meter (Hach Company, CO, USA). Thirteen milliliters of 0.22 μm filtered (Whatman International Ltd, Kent, UK) water was collected in 15 mL falcon tubes and frozen for later geochemical analysis. NH_4_, NO_2_/NO_3_, and PO_4_ measurements of the overlying pond filtrate from selected samples were carried out at the University of Waikato using an Aquakem 200CD following the manufacturers instructions (Thermo Fisher Scientific, Waltham, USA). Elemental analysis was performed on each sample by inductively coupled plasma mass spectrometry (ICP-MS) using a Mass Spectrometer ELAN® DRC II (PerkinElmer Inc., Münster, Germany). To prepare samples for ICP-MS, 0.22 μm prefiltered pond water was diluted 1:50 with Milli-Q water (Millipore, Billerica, MA, USA). Once diluted, samples were acidified with 2% HN0_3_(Extra pure Nitric Acid, Ajax Finechem, NSW, Australia). Differences in Aluminum concentration between sites were investigated using Tukeys Honest Significant Difference Test (in R). In Primer 6 (Clarke and Gorley, 2006) geochemical data was transformed using a square root followed by a log (X+1) transformation and normalization. Two-dimensional ordinations using non-metric multidimensional scaling (NMDS) was performed based on a Euclidean distance matrix to represent the relative distances between individual ponds.

### DNA extraction

DNA was extracted from 0.5 g ± 0.1 g of individual sediment sections using a modified bead-beating method (Coyne et al., 2001). Briefly, sediment was added to 0.5 g each of 0.1 mm and 2.5 mm silica-zirconia beads. To each sample 270 μL of phosphate buffer (100 mM NaH_2_PO_4_) and 270 μL of SDS lysis buffer (100 mM NaCl, 500 mM Tris pH 8.0, 10% SDS) were added and samples were horizontally shaken on a Vortex Genie 2 (MO BIO Laboratories Inc, Carlsbad, CA, USA) for 15 min. Samples were centrifuged at 12,500 rpm for 30 s and 180 μL of cetyltrimethylammonium bromide-polyvinylpyrrolidone (CTAB) extraction buffer (100 mM Tis-HCl, 1.4 M NaCl, 20 mM EDTA, 2% CTAB, 1% polyvinylpyrrlidone and 0.4% β-mecaptoethanol) was added. Samples were vortexed for 10 s prior to incubation at 60°C and 300 rpm for 30 min on a rocking bed. Samples were centrifuged at 12,500 rpm for 30 s and then 350 μL of chloroform/isoamyl alcohol (24:1) was added. Samples were again vortexed for 10 s and centrifuged for 5 min at 12,500 rpm. The aqueous phase was transferred to a new eppendorf tube then 500 μL of chloroform/isoamyl alcohol (24:1) was added. Samples were vortexed and left on a rocking bed HulaMixer (Invitrogen, Carlsbad, CA, USA) for 20 min. Samples were centrifuged for 5 min at 13,500 rpm, the aqueous phase was removed and 10 M ammonium acetate was added to the samples to achieve a final concentration of 2.5 M. The samples were vortexed and centrifuged for 5 min at 13,500 rpm. The aqueous layer was removed to a new tube and 0.54 volumes of isopropanol was added and mixed. Samples were left overnight at −20°C then centrifuged for 20 min at 13,500 rpm. The supernatant was removed, the pellet washed with 1 mL of 70% AR grade ethanol and centrifuged for 1 min at 13,500 rpm. Ethanol was removed and DNA was re-suspended in 30 μL of sterile TE then quantified using the Qubit 2.0 Florometer (Invitrogen). The four individual sectioned samples from each core were diluted to 10 ng/μL, then 10 μL of each was pooled and frozen at −20°C until use.

### ARISA community fingerprinting and analysis

Automated Ribosomal Intergenic Spacer Analysis (ARISA) DNA fingerprinting (Fisher and Triplett, 1999) was utilized to resolve relative structure between bacterial communities as a preliminary comparison between ponds and sites (Archer et al., 2014). Briefly, from each sample the bacterial intergenic spacer region (ISR) in the rRNA operon was amplified using PCR primers ITSReub-Hex (5′-GCCAAGGCATCCACC-3′) and ITSF (5′-GTCGTAACAAGGTAGCCGTA-3′) according to Cardinale et al. (2004). All ARISA PCR reactions were run in triplicate on a Bio-Rad DNA Engine® (PTC-200) Peltier Thermal Cycler (Bio-Rad Laboratories Inc, Hercules, CA). Thermal cycling conditions were: 94°C for 5 min, then 30 cycles of 94°C for 45 s, 55°C for 1 min, 72°C for 2 min, and a final extension of 72°C for 7 min. Once amplified, all triplicate PCR reactions were resolved on a 1% agarose gel to ensure amplification success then pooled. Amplicons were diluted 1:20 in Gibco ultrapure water and fragment lengths were resolved on a 3130XL DNA sequencer (Applied Biosystems, New York, USA) using Liz-1200 internal size standard at the University of Waikato DNA Sequencing Facility.

ARISA fingerprints were processed with an informatics pipeline (modified from Abdo et al., 2006; Sokol et al., 2013). Peaks exceeding 200 fluorescence units, greater than 50 bp and less than 1200 bp were accepted as true peaks. The remaining peaks were used to calculate model parameters for a log-normal distribution. Iteratively, peaks with an area exceeding the 99.9% cumulative distribution of the calculated log-normal distribution for noise were accepted as true peaks. Peaks were binned into ARISA Fragment Lengths (AFLs) within 5 bp of one another. The resulting data matrix was analyzed using a combination of Primer 6 (Clarke and Gorley, 2006) and R for statistical analysis (R Core Team, 2013). In Primer 6, beta diversity was investigated using a resemblance matrix created based on the Bray Curtis community dissimilarities. These were examined in a two-dimensional ordination using non-metric multidimensional scaling (NMDS). ANOSIM analyses were performed on the resemblance matrix to test specific hypotheses formed from interpretation of MDS plots.

### DNA pyrosequencing

The V5-V6 hypervariable region of the 16S rRNA gene was utilized to identify variation in bacterial community diversity and structure using primers and conditions detailed in Archer et al. (2014). Briefly, triplicate 30 μL PCR reactions were run for each sample using un-adapted primers Tx9F (5′-GGATTAGAWACCCBGGTAGTC-3′) and 1391R (5′-GACGGGCRGTGWGTRCA-3′). The triplicates were pooled then gel extracted on a 2% TAE agarose gel stained with “SYBR Safe” and DNA was retrieved using the UltraClean 15 (MoBio, Inc, Carlsbad, USA) DNA Purification Kit as per manufacturers instructions. A second round of triplicate PCR was run as above but with only 10 cycles and using 25 ng of the purified DNA from the previous step per reaction (milli-Q H_2_O volume adjusted accordingly). The primers used were adapted for one-way reads as per manufacturers instructions, including unique MID identifiers for each sample [BacX-Tx9F (5′-CCATCTCATCCCTGCGTGTCTCCGACTCAG-MID-GGATTAGAWACCCBGGTAGTC-3′) and BacB-1391R (5′-CCTATCCCCTGTGTGCCTTGGCAGTCTCAG-GACGGGCRGTGWGTRCA-3′)]. A second gel extraction was performed as above. Samples went through a final cleanup step using the Agencourt AMPure XP system (Beckman Coulter Genomics, Danvers, MA, USA) as per the manufacturers instructions. Sample DNA content was quantified using a Qubit Fluorometer and diluted to 200 pg/μL. The DNA concentration and quality verification was performed using a 2100 Bioanalyzer (Agilent Technologies, Waldbronn, Germany) and then diluted to 1 × 10^9^ molecules/μL. The diluted amplicons were mixed together in the desired proportions to create the 1 × 10^9^ amplicon pool. Sequencing was performed using the GS Junior Titanium emPCR Kit (Lib-L), the GS Junior Titanium Sequencing Kit, PicoTiterPlate Kit and GS Junior System according to the manufacturers instructions (Roche 454 Life Sciences, Branford, CT, USA).

The 454 amplicon pyrosequencing data was processed using AmpliconNoise v1.0 for quality filtering, denoising and chimera removal (Quince et al., 2011). Briefly, raw flowgrams (sff files) with perfectly matching primer and barcode sequences were filtered for a minimum flowgram length of 360 cycles (including primer and barcode sequences) before the first noisy signal (i.e., 0.5–0.7 or no signal in all four nucleotides). All flowgrams were then truncated at 360 bases and clustered to remove sequencing noise using PyroNoise (Quince et al., 2009, 2011). Noise introduced by PCR was removed using SeqNoise (Quince et al., 2011), and PCR chimeras were removed using Perseus (Quince et al., 2011). Resulting de-replicated sequences from Perseus were processed using Mothur 1.17.0 (Schloss et al., 2009) to create a unique sequence and names file. Pairwise alignments and distance were calculated using Espirit (Sun et al., 2009). Mothur was then used to cluster sequences into operational taxonomic units (OTUs) defined at the average neighbor Jukes-Cantor distance of 0.05 (OTU_0.05_). Rank-abundance data were generated for each sample. To evaluate the effects of uneven sampling depths in this investigation subsampling was conducted on the dataset with the sub.sample command in mothur. As subsampling resulted in little significant change to the observed community structure maximum diversity across all samples was retained.

For phylogenetic assignments, representative sequences of all identified OTU_0.05_ were analyzed using the Classifier function provided by the Ribosomal Database Project (RDP) Release 10, Update 15 (Wang et al., 2007). Taxonomic assignment threshold was set at 80%. To gain a visual representation of individual sample community similarity based on sequencing data a dendrogram was created in Primer 6 using a resemblance matrix based on the Bray Curtis community similarities. BEST analysis, a trend correlation tool in Primer 6 (Clarke and Gorley, 2006) that identifies the maximum rank correlation with 99 permutations between the normalized geochemical data and the pyrosequencing data, was conducted to investigate geochemical drivers to community structure.

DNA sequences generated in this study are deposited in the European Nucleotide Archive (ENA) under accession number PRJEB8342.

## Results

### Pond geochemistry

**Figure 2:**
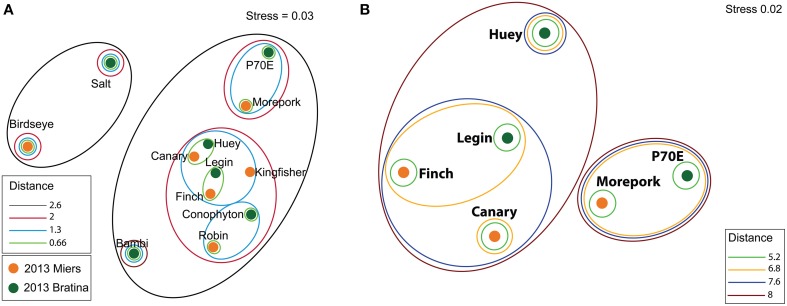
**MDS ordinations of sediment geochemical profiles. A Euclidean distance matrix was calculated using geochemical data that was square root, log (X+1) transformed and normalized. In field collected preliminary geochemistry (pH, DO, and conductivity) from all ponds (A)**. Preliminary geochemistry, ICP-MS, and nutrient values of selected subset of ponds (Supplementary Table 1) **(B)**.

*In situ* geochemistry varied across all ponds with DO values ranging from 11.6 to 20.2 ppm, conductivity from 0.6 to 40.8 mS cm^−1^ and pH from 8.8 to 10.6 (Table 1). The relationship between in-field collected geochemistry (pH, DO and conductivity) of all ponds is represented in a non-metric MDS plot (Figure 2A). Based on this plot, six ponds, three from each location, were identified for higher resolution geochemical (nutrient and elemental) and genetic analyses. Comparisons of geochemical profiles including nutrient and elemental data (Supplementary Table 1) are represented by a non-metric MDS plot (Figure 2B), in which ponds between locations clustered closer together than within locations.

### Benthic microbial community structure in meltwater ponds

**Figure 3:**
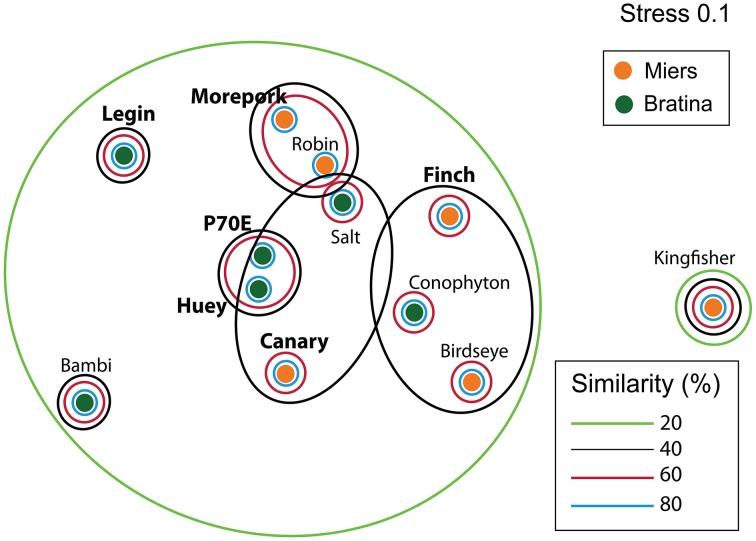
**Non-metric multidimensional scaling (NMDS ordinations of bacterial ARISA community compositions based on Bray Curtis distances (Stress = 0.1)**. Samples collected in January 2013 from Bratina Island (6 samples) and the Miers Valley (6 samples). Larger bold names represent samples selected for high-throughput sequencing.

**Figure 4:**
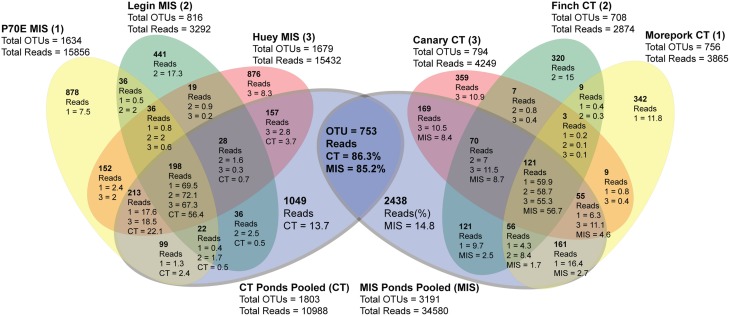
**Summary of 454 sequencing data (distance = 0.05) representing shared OTUs (in bold) and reads (%) from individual ponds and pooled data between sample locations**. MIS ponds pooled (MIS) represents all P70E, Legin and Huey reads to compare with individual CT ponds; CT ponds pooled represents all Morepork, Finch and Canary reads to compare with individual MIS ponds.

**Figure 5:**
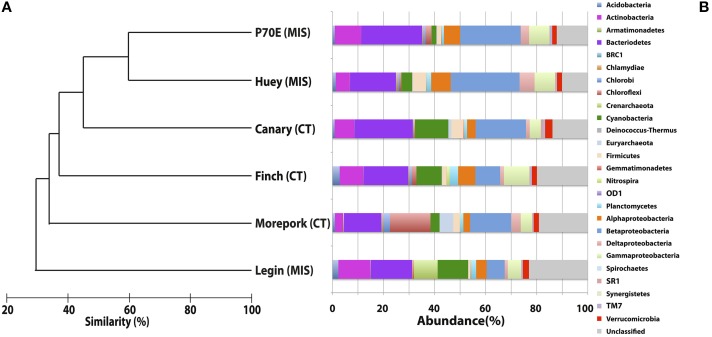
**MAQ[-34mm]Q1Bray-Curtis Tree and Phylum-level distribution of 16S rRNA OTUs**. Bray-Curtis tree calculated from total OTU_0.05_ compositions with no transformation to visualize total relative spatial/temporal similarities between water columns **(A)**. Phylum-level distribution of bacterial 16S rRNA OTUs_0.05_ assigned using the Ribosomal Database Project (RDP) Release 10, Update 15 Classifier, assignment confidence threshold >80% **(B)**.

Inter-pond benthic bacterial ARISA community structure based on a non-metric MDS plot (Figure 3) had a broad distribution with little clustering based on location (Bratina or Miers). Six benthic samples (three from each location) were selected for high-throughput sequencing (Supplementary Table 2). A venn diagram was created to represent unique and shared OTUs and reads from pooled data between locations (Bratina 2013 and Miers Valley 2013) and between individual ponds. A total of 753 shared OTUs represented 86.3 and 85.2% of total reads in the Miers Valley and Bratina Island respectively (Figure 4). Benthic community structure of individual ponds based on OTU abundance was heterogeneous (<50% similarity between all ponds except P70E and Huey), although phyla compositions appeared similar (Figure 5). The large proportion of shared sequences and similar phyla compositions but low community structure similarity between ponds resulted from variable abundances of dominant OTUs (Table 2). The most biologically similar ponds among the six ponds examined were P70E and Huey, however, multiple variations in phyla and OTU abundance were identified (Figure 5, Table 2). The relative abundances of *Actinobacteria* (10.2 vs. 5.5%) and *Bacteroidetes* (23.6 vs. 17.8%) were higher in P70E, whereas *Proteobacteria* (41.8 vs. 49.0%), *Firmicutes* (1.6 vs. 4.2%), and *Cyanobacteria* (1.9 vs. 4.2%) were less abundant in P70E. This variation extended to the abundance of individual OTUs (Table 2). OTUs unique to individual ponds represented 7.5 to 17.3% of total pond reads however accounted for 45.2 to 54.0% of total OTUs from each pond (Figure 4).

**Table 2:** **Heatmap of abundance of the 15 most abundant OTUs based on sequencing analysis**.

**OTU**	**Miers Valley**			**Bratina Island**				**Pooled**	
	**Morepork (%)**	**Canary (%)**	**Finch (%)**	**Huey (%)**	**Legin (%)**	**P70E (%)**	**Total (%)**	**Pooled Bratina (%)**	**Pooled Miers (%)**
1	4.58	14.64	4.24	14.98	3.22	8.70	7.19	6.72	7.82
2	5.77	0.49	0.07	4.70	0.12	9.38	2.93	3.55	2.11
3	0.62	11.41	2.33	2.02	2.92	2.05	3.05	1.75	4.79
4	1.11	0.14	1.15	1.71	0.73	5.81	1.52	2.06	0.80
5	2.10	2.40	4.63	2.46	2.16	3.32	2.44	1.98	3.04
6	3.10	1.93	2.47	3.39	0.79	2.85	2.08	1.76	2.50
7	0.93	1.01	1.57	3.91	0.70	2.24	1.48	1.71	1.17
8	1.37	0.33	0.24	3.66	0.24	1.81	1.09	1.43	0.65
9	0.88	0.75	1.91	1.54	0.49	2.80	1.20	1.21	1.18
10	0.88	1.60	3.27	1.42	2.25	1.78	1.60	1.36	1.92
11	0.67	1.84	2.37	1.46	1.18	1.98	1.36	1.16	1.62
12	0.00	4.14	0.14	0.41	1.37	0.32	0.91	0.52	1.43
13	0.21	0.28	0.03	1.89	1.37	0.88	0.67	1.04	0.17
14	11.25	0.12	0.24	0.15	0.00	0.89	1.81	0.26	3.87
15	0.39	5.70	0.00	0.25	2.00	0.16	1.21	0.60	2.03
Total	33.87	46.79	24.67	43.97	19.53	44.97	30.54	27.12	35.11
	**NCBI Accesssion**	**Identity (%)**	**Phylum**	**Class**	**Organism**		**Description**		
1	KM035974.1 GQ332345.2	100 100	Proteobacteria	Betaproteobacteria	Xylophilus sp Variovorax sp.		Waterfall, Korea Rice paddy fields Soil, South Korea		
2	NR_044555.1 NR_074417.1	99 99	Proteobacteria	Betaproteobacteria	Thiobacillus thiophilus Thiobacillus denitrificans		Aquifer sediments, Germany Obligately chemolithoautotrophic, facultatively anaerobic		
3	JX287872.1 KF318413.1	99 99	Bacteroidetes	Flavobacteria	Flavobacterium oncorhynchi Flavobacterium sp.		Fish associated, Mishigan, USA Soil, Kyrgystan		
4	FR691443.1 AJ441008.1	98 98	Bacteroidetes	Flavobacteria	Gelidibacter algens Antarctic bacterium R-9217		Forlidas pond (Pensacola Mountains) and Lundstrom (Shackleton Range), Antarctica Microbial Mats, 10 Antarctic Lakes		
5	KF923805.1 KC921170.1	99 99	Proteobacteria	Gammaproteobacteria	Thermomonas sp. Thermomonas brevis		Soil, China Soil, China		
6	AB769197.1 GQ369139.1	98 98	Proteobacteria	Betaproteobacteria	Ideonella sp. Ideonella sp.		Rice paddy fields, Japan Rice paddy fields, South Korea		
7	KF441682.1 JQ692104.1	99 99	Proteobacteria	Alphaproteobacteria	Rhodobacter sp. Rhodobacter megalophilus		Urgeirica mine, Portugal water and sediments Swamp water, South Korea		
8	NR_115066.1 NR_102510.1	98 96	Proteobacteria	Deltaproteobacteria	Desulfopila inferna Desulfocapsa sulfexigens		Tidal flat sediments, Germany Marine		
9	FJ196000.1 NR_109527.1	100 99	Bacteroidetes	Sphingobacteria	Algoriphagus sp. Algoriphagus chungangensis		Marine sediments, Antarctic ocean Tidal flat sediment, South Korea		
10	JN848793.1 NR_074595.1	99 97	Bacteroidetes	Sphingobacteria	Terrimonas sp. Niastella koreensis		Soil, South Korea Soil, Psychrophilic, South Korea		
11	NR_041633.1 NR_112714.1	100 99	Actinobacteria	Actinobacteria	Ilumatobacter fluminis Ilumatobacter coccineus		Estuary sediment, USA Marine Sand, Japan		
12	KF029609.1 AY493582.1	99 97	Cyanobacteria	Oscillatoriophycideae	Uncultured cyanobacterium clone Phormidesmis priestleyi		Freshwater microbial mat, meltwater pond, Antarctic peninsula Cyanobacteria, Antarctica		
13	AJ293105.1 AY038032.1	99 99	Cyanobacteria	Nostocales	Anabaena solitaria Anabaena flos-aquae		Cyanobacteria, Nostoc, Antarctica Cyanobacteria		
14	EU283576.1 NR_041354.1	99 93	Chloroflexi	Anaerolineae	Uncultured Chloroflexi bacterium Bellilinea caldifistulae		Anderson lake, USA Thermophilic digester sludge		
15	KC633966.1 KC633965.1	100 100	Cyanobacteria	Oscillatoriophycideae	Microcoleus sp. Microcoleus sp.		Dump soil, USA Antarctica		

*The color intensity represents a larger fraction of total sequences. Values representative of total reads corresponding to these 15 OTUs in each sample are in Purple, location based totals of each OTU (Green) and individual sample (Red high, Blue low). NCBI accession numbers, classifications and available information of sample origin are also included*.

A total of 23 phyla were identified across all ponds sampled (Figure 5), with variable relative abundance between ponds, however those representing >1% of total reads from all samples were present in all samples (*Proteobacteria, Bacteroidetes, Acidobacteria, Chloroflexi, Cyanobacteria, Euryarchaeota, Firmicutes, Planctomycetes*, and *Verrucomicrobia*). *Chloroflexi* (6 to 1.2%), *Crenarchaeota* (<0.1 to 1.2%), *Euryarchaeota* (2.2 to 0.01%), and *Cyanobacteria* (8.8 to 3.8%) exhibited the greatest phyla differences between pooled Miers Valley and Bratina Island benthic communities. Some phyla were detected at particularly high relative abundance in individual ponds, such as *Chloroflexi* in Morepork, and *Crenarchaeota* in Legin pond (almost 10-fold higher than from any other pond). Sequences affiliated with *Bacteroidetes* and *Proteobacteria* were dominant across all ponds, accounting for 14.6–23.6% and 19.3–49.0% of sequences, respectively. Variability between samples was also examined at class level for *Proteobacteria*, the dominant class being *Betaproteobacteria* followed by *Gammaproteobacteria* and *Alphaproteobacteria*.

### Geochemical drivers of community structure

BEST analysis was performed to examine how geochemistry affects benthic community structure, measured using pyrosequencing, between ponds. Potassium, sodium and cobalt (Supplementary Table 1) were the strongest explanatory variables to total community structure (Spearman's correlation coefficient value of 0.632, 0.436, and 0.428 respectively) and to cosmopolitan OTUs (those present in at least one pond at each location) (Spearman's correlation coefficient-value of 0.607, 0.368, and 0.392). BEST analysis of unique OTUs (those only present at one location) identified aluminum, uranium and manganese as the strongest explanatory variables to community structure (Spearman's correlation coefficient-value of 0.806, 0.435, and 0.415 respectively). BEST analysis of unique OTUs based on the subsampled dataset resulted in nearly identical results (Spearman's correlation coefficient-value of 0.806, 0.435, and 0.415 for Al, U and Mg respectively). A Tukey Honest significance difference test between locations confirmed a significantly higher concentration of Aluminum in the MIS ponds (*P* = 0.006).

## Discussion

The meltwater ponds included in this study represent the high degree of geochemical heterogeneity typically found in Antarctic meltwater ponds (Healy et al., 2006; Wait et al., 2006; Lyons et al., 2012; Hawes et al., 2013). The study sites include two distinct underlying substrate types, which are known to influence cyanobacterial mat structure (de los Rios et al., 2004). The sediments from the McMurdo Ice Shelf (MIS) at Bratina Island are marine derived while the Coastal Terrestrial (CT) ponds in the Miers Valley are derived from glacial weathering of bedrock (Kellogg and Kellogg, 1987, 1988; McLeod et al., 2009; Cary et al., 2010). Similar geochemical pond profiles were identified between locations, with little coherence between ponds from the same location. The geochemical similarity between locations is surprising as many meltwater ponds reside in closed basins, their geochemical properties reflective of their immediate environment often exhibiting marked variations in elemental composition within (Matsumoto et al., 1992; Hawes et al., 2013), and particularly between sites (Healy et al., 2006).

Community DNA profiling of benthic bacterial communities using ARISA revealed little clustering based on location. High throughput pyrosequencing of representative ponds identified a diverse community, yet more than 85% of total sequences from all samples were shared between locations, suggesting movement and redistribution of microorganisms at a regional scale. Given the close proximity (<40 km) between Bratina Island and the Miers Valley, aeolian transportation of microorganisms between locations likely takes place. This has been demonstrated for invertebrates and *Cyanobacteria* throughout the McMurdo Dry Valleys (Nkem et al., 2006; Wood et al., 2008) and suggested for Bacteria across large (>800 km) distances along the Transantarctic Mountains (Sokol et al., 2013). Katabatic winds in the Miers Valley move down from the Polar Plateau onto the ice shelf (Bottos et al., 2014), where storms transport large volumes of dust, mostly from a southerly direction (Atkins and Dunbar, 2009; Dunbar et al., 2009). Bratina Island is located northeast of Miers Valley (Figure 1) and receives its strongest winds from the southeast or southwest (Hawes et al., 2013), providing a mechanism for transportation.

Variations to community structure between pond benthic zones was identified, even between Huey and P70E, the most biologically similar ponds in this study. Community variation was manifested primarily in the abundance of several key phyla and OTUs, as has been seen previously (Bowman et al., 2000), and was likely the result of the highly variable geochemistry of each pond (Jungblut et al., 2005). The small number of pond specific sequences identified in this study represented a large localized diversity. Although of low abundance at the sampling time point a number of these taxa may have been more significant throughout the year due to the extreme seasonality experienced by these ponds (Hawes et al., 1999; Laybourn-Parry, 2002).

The depth of sequencing coverage in this study resulted in the detection of 23 phyla, a far greater diversity than previously reported in comparable ecosystems (Bowman et al., 2000; VanTrappen et al., 2002; Sjoling and Cowan, 2003; Peeters et al., 2012; Tang et al., 2013). Although there was a large core of shared sequences, inter-site variation in community structure (abundance of individual phyla and OTUs) was identified, as seen between *Crenarchaeota* and *Euryarchaeota*. *Crenarchaeota*, a highly abundant phylum in marine environments (Karner et al., 2001) was present at a ten-fold higher abundance at Bratina Island, consistent with previous findings (Sjoling and Cowan, 2003). *Euryarchaeota* abundance was 200 fold higher in the Miers Valley, and was previously the only *Archaea* identified in a study of six Antarctic Dry Valley lakes (Vestfold Hills, Princess Elizabeth Land) (Bowman et al., 2000). This shows that although the bulk microbial community is shared between locations there are microorganims closely linked to a specific biome.

Several high abundance phyla were ubiquitous in all pond sediments in this and previous studies, the most abundant being *Proteobacteria* (Bowman et al., 2000; VanTrappen et al., 2002; Sjoling and Cowan, 2003; Peeters et al., 2012; Tang et al., 2013). Similar to past studies, *Alpha* and *Gammaproteobacteria* were present in all ponds (Bowman et al., 2000; Peeters et al., 2012; Sjoling and Cowan, 2003; Tang et al., 2013; VanTrappen et al., 2002), however *Betaproteobacteria*, most abundant in this study (Bowman et al., 2000; Sjoling and Cowan, 2003) and *Deltaproteobacteria* (VanTrappen et al., 2002; Peeters et al., 2012) were not detected in all studies. *Bacteroidetes* was the only other phyla present across all studies with *Actinobacteria, Firmicutes, Planctomycetes, Verrucomicrobia, Acidobacteria*, and *Chloroflexi* intermittently identified at low abundance in the other studies, consistent with our findings (Bowman et al., 2000; VanTrappen et al., 2002; Sjoling and Cowan, 2003; Peeters et al., 2012; Tang et al., 2013). These results indicate that previous investigations had likely detected the most abundant portion of the ecosystem, however, given the high number of previously undetected phyla, a large part of the diversity was missed.

*Cyanobacteria* were poorly represented in the pyrosequencing data, a result of deliberate low PCR primer matching (<1%) to known cyanobacterial sequences (https://rdp.cme.msu.edu/probematch/search.jsp). However, as this bias would exert an equal effect between samples, cyanobacterial sequence abundances are comparable within this study. Variable abundances of cyanobacterial OTUs were identified between locations and within ponds. This was likely related to selection pressures caused by source material (for example grain size) (de los Rios et al., 2004) and conductivity (variable within ponds) (Jungblut et al., 2010). The continual layering of aeolian dust causes surface cyanobacterial mats to be covered and thus form sediments (Squyres et al., 1991), therefore difference in cyanobacterial mat communities is likely to have a significant impact on the formation of the underlying sediment communities. Cyanobacterial mats, with a complicated associated bacterial community (Moorhead et al., 2003; Barrett et al., 2006; Wood et al., 2008), are a possible vector for microbial transportation between the Miers Valley and Bratina Island (Vincent, 2000). These mats are have been shown to be capable of distribution throughout the McMurdo Dry Valleys and remain viable (de los Rios et al., 2004), although a recent study suggests that distribution distances for *Cyanobacteria* in Antarctica may be limited to <3 km (Sokol et al., 2013).

BEST analysis identified potassium, an essential intracellular cation in most bacteria (Epstein, 2003; Malek et al., 2012), as most significantly correlated with differences in the cosmopolitan community between ponds (Supplementary Table 1). A BEST analysis on location unique sequences provided a distinct set of correlated variables the strongest of which, more significant to structuring the unique community than Potassium to the cosmopolitan community, was Aluminum. Although poorly soluble in alkaline water aluminum is an abundant metal in the Earth's crust with recognized anti microbial effects (Pina and Cervantes, 1996). Higher aluminum concentrations in all MIS ponds is likely a contributing factor to community variation between locations. The variable concentration of these elements in the ponds is likely reflective of differences in local geology (Campbell and Claridge, 1977).

Although habitat type is known to affect community structure of Antarctic microbial mats (Peeters et al., 2012), the communities of the coastal terrestrial (Miers Valley) and MIS (Bratina Island) ponds appear to share the majority of the dominant OTUs, likely due to close geographic proximity and favorable wind direction between locations. Variation in communities between ponds and locations was related to differences in major OTU abundances and the presence of pond specific diversity likely driven by local geochemistry. These factors should be taken into account when investigating regional community variation in future studies.

### Conflict of interest statement

The authors declare that the research was conducted in the absence of any commercial or financial relationships that could be construed as a potential conflict of interest.
