# Supplementary material for: Benthic microbial communities of coastal terrestrial and ice shelf Antarctic meltwater ponds
Source: Front Microbiol. 2015 May 27;6:485. doi: 10.3389/fmicb.2015.00485 (PMC4444838; doi:10.3389/fmicb.2015.00485)
Supplement: Supplementary file 1 [file Table1.DOC]

**Supplementary Table 1:** Complete geochemistry dataset including ICPMS and nutrient analysis. NO_2_ and NO_3_ were below detection limit for all samples so are not presented in the table
