# Supplementary material for: Benthic microbial communities of coastal terrestrial and ice shelf Antarctic meltwater ponds
Source: Front Microbiol. 2015 May 27;6:485. doi: 10.3389/fmicb.2015.00485 (PMC4444838; doi:10.3389/fmicb.2015.00485)
Supplement: Supplementary file 2 [file Table2.DOC]

**Supplementary Table 2:** Summary of pyrosequencing statistics of individual sediment samples including number of unique sequences (OTUs per sample), total sample reads, number of OTUs found at a single copy per sample and Goods Coverage estimator for all samples.
